# QCT-based finite element prediction of pathologic fractures in proximal femora with metastatic lesions

**DOI:** 10.1038/s41598-019-46739-y

**Published:** 2019-07-16

**Authors:** Emir Benca, Alexander Synek, Morteza Amini, Franz Kainberger, Lena Hirtler, Reinhard Windhager, Winfried Mayr, Dieter H. Pahr

**Affiliations:** 1 Department of Orthopedics and Trauma Surgery, Medical University of Vienna, 1090 Vienna, Austria; 2 Institute for Lightweight Design and Structural Biomechanics, TU Wien, 1060 Vienna, Austria; 3 Department of Radiology, Medical University of Vienna, 1090 Vienna, Austria; 4 Division of Anatomy, Center for Anatomy and Cell Biology, Medical University of Vienna, 1090 Vienna, Austria; 5 Center for Medical Physics and Biomedical Engineering, Medical University of Vienna, 1090 Vienna, Austria

**Keywords:** Bone metastases, Experimental models of disease

## Abstract

Predicting pathologic fractures in femora with metastatic lesions remains a clinical challenge. Currently used guidelines are inaccurate, especially to predict non-impeding fractures. This study evaluated the ability of a nonlinear quantitative computed tomography (QCT)-based homogenized voxel finite element (hvFE) model to predict patient-specific pathologic fractures. The hvFE model was generated highly automated from QCT images of human femora. The femora were previously loaded in a one-legged stance setup in order to assess stiffness, failure load, and fracture location. One femur of each pair was tested in its intact state, while the contralateral femur included a simulated lesion on either the superolateral- or the inferomedial femoral neck. The hvFE model predictions of the stiffness (0.47 < R^2^ < 0.94), failure load (0.77 < R^2^ < 0.98), and exact fracture location (68%) were in good agreement with the experimental data. However, the model underestimated the failure load by a factor of two. The hvFE models predicted significant differences in stiffness and failure load for femora with superolateral- and inferomedial lesions. In contrast, standard clinical guidelines predicted identical fracture risk for both lesion sites. This study showed that the subject-specific QCT-based hvFE model could predict the effect of metastatic lesions on the biomechanical behaviour of the proximal femur with moderate computational time and high level of automation and could support treatment strategy in patients with metastatic bone disease.

## Introduction

Today, life expectancy of cancer patients is increasing. As a result, there is a higher prevalence of metastatic bone disease that affects patients with a rather good prognosis^1^. Metastatic cancer with skeletal involvement can result in a pathologic bone fracture, often in absence of a traumatic event and during everyday activities. Despite multiple treatment options, surgical intervention is often required due to poor bone healing and the patient’s limited life expectancy^2^. The proximal femur is among the most affected anatomical sites^3^. When diagnosed, bone metastases present a great challenge in choosing the appropriate treatment strategy. Prophylactic fixation is generally preferred to pathologic fracture and its subsequent treatment^4,5^. However, current clinical guidelines and methodologies for fracture risk prediction, such as the Mirels’ scoring system^6^ are not specific enough, particularly in non-impending fracture prediction^7^.

In summary, by defining patient-specific fracture risk, patients with an impending pathologic fracture would benefit from a prophylactic intervention, while patients with a non-impending fracture could be spared from unnecessary treatment.

Assessing strength of a metastatic bone requires information on its geometry, three-dimensional distribution of density^8^, the size^9^, shape^10^, site^11^, and nature of the lesion^6,12^, as well as on the patient’s loading regimen and activity level.

The finite element analysis (FEA) is a computer simulation method which has been recognized as a powerful tool for bone fragility prediction^13–20^. FEA considers both, the individual geometric and densitometric parameters retrieved from computed tomography imaging. In homogenized continuum level voxel based FE (hvFE) models, voxels are directly converted to hexahedral elements with specific homogenized material properties depending on local bone density.

Several authors have applied FEA to predict fracture loads of femora with metastatic lesions and reported good correlations to *in vitro* testing^21–25^. However, these data have often been based on a small number of specimens, mostly with one specimen per specific site of the metastatic lesion. More importantly, most modeling has been performed using fine finite element meshes associated with high computational resources and longer analysis time. A single finite element analysis of a femoral fracture was recently estimated to take four to ten hours^24,26^. Furthermore, the model generation and analysis requires profound technical knowledge of the operator and, thus, specially trained personnel becomes inevitable. As a result, increasing the level of automation is mandatory to make this method time- and cost-effective, and finally, to include it in the clinical routine.

The objective of the present study was to introduce QCT-based finite element models to predict patient-specific pathologic fracture loads of proximal femora in a clinical context. We hypothesized that the FE models allow predicting the fracture load of pathologic femora just as well as fracture load of intact femora. Model validation was performed on a large number of specimens (n > 30) using previously collected *in vitro* experimental data. To meet clinical demands, patient-specific models were generated with a high level of automation and simplified to minimize analysis time.

## Methods

### Study outline

**Figure 1:**
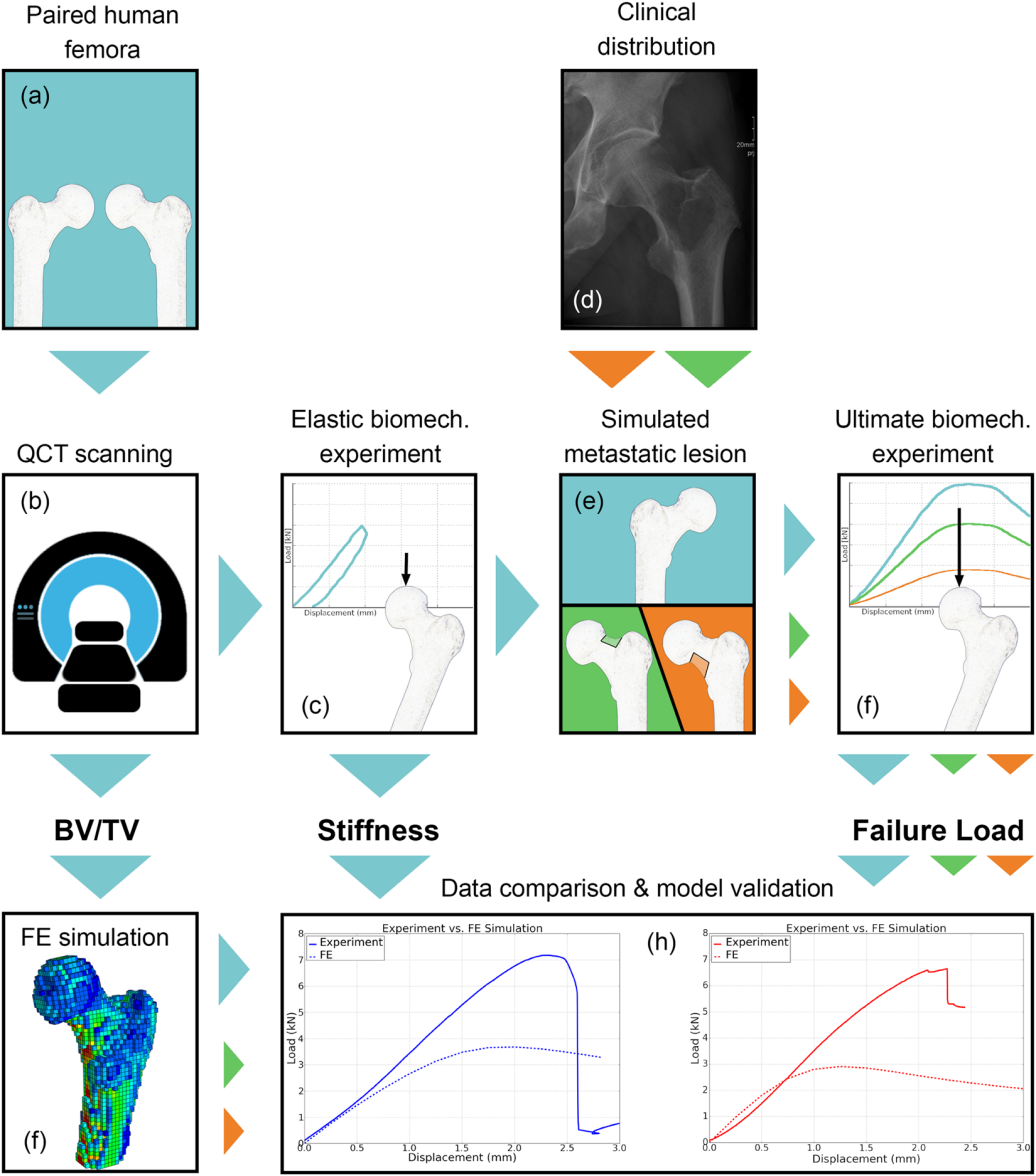
Graphical abstract of the project: Thirty-two paired femora (**a**) underwent QCT scanning (**b**) and elastic biomechanical experiments to determine stiffness (**c**). Based on clinical distribution in patients who suffered a pathologic fracture (**d**), metastatic lesions were simulated in either the superolateral or inferomedial neck in one femur of each pair (**e**), followed by a second QCT scan and stiffness test. All femora were then subjected to an ultimate biomechanical test to determine failure load^11^ (**f**). A FE model was generated based on geometry and bone density distribution (BV/TV) retrieved from QCT images (**g**). Experimental data were used to evaluate the QCT-based FE model (**h**).

As illustrated in the graphical abstract, (Fig. 1) first, QCT image sequences of human femoral specimens were acquired. Thereafter, the specimens were subjected to mechanical compression in stiffness- and failure load tests. Tests were performed on intact specimens as well as on specimens with simulated metastatic lesions. The results of the mechanical experiments were published previously^11^. Nonlinear hvFE models were generated from QCT images and validated with data assessed in mechanical experiments on specimens under careful consideration of experimental boundary conditions. The study was approved by the Ethics Committee of the Medical University of Vienna (1400/2013). The specimens were obtained from voluntary donors to the Center for Anatomy and Cell Biology, Medical University of Vienna, who consented in written form during their lifetime to the use of their bodies for research and education. All study procedures were conducted in accordance with the guidelines approved by the Ethics Committee and the Declaration of Helsinki.

### QCT scanning and mechanical testing

For the direct validation of the FEA, the authors used QCT-images from anatomic specimens, previously loaded in mechanical experiments. For a detailed description of the experiments, the reader is referred to this previous work^11^. In short, a total of 32 paired fresh frozen human femoral anatomic specimens (9 female, 7 male, mean age 79.7 ± 10.7 years) were used. The sample size and age were restricted by limited availability of human anatomic specimens. One femur of each pair remained intact, while the contralateral femur was assigned to the metastatic group, in which a simulated metastatic lesion was created on either the superolateral- or inferomedial femoral neck. One specimen from the group with superolateral lesions was excluded due to severe head necrosis. The simulated lesions occupied one third of the cortical circumference over the whole length of the specified neck site including the underlying cancellous bone and were created using a milling-cutter. These regions were chosen based on retrospective clinical data of patients who suffered a pathologic fracture^11^.

The most distal part of each femur was dissected. QCT image sequences were acquired with the Brilliance CT 64-channel scanner (Philips, Amsterdam, Netherlands) with a voxel size of 0.33 mm × 0.33 mm × 1 mm. The samples were scanned in 0.9% saline solution on top of a calibration phantom, BDC Phantom (QMR GmbH, Möhrendorf, Germany).

Additionally, the centrum-collum-diaphyseal (CCD) angle was measured from original uncropped QCT image sequences using ImageJ (developed by Wayne Rasband at NIH). Also, the volumetric bone mineral density (vBMD) and bone mineral content (BMC) were retrieved from the scans for the masked proximal part of each specimen before and after creating the lesion in one region of interest including head, neck, trochanter, and intertrochanteric region, using Medtool v.4.0 software (Dr. Pahr Ingenieurs e.U., Pfaffstätten, Austria).

Following imaging, the specimens were embedded, rigidly fixed in the experimental setup, and loaded in a quasi-static mode, mimicking one-legged stance. One-legged stance is not representative for all real life fracture mechanisms, which are inherently different from one patient to another. Nevertheless, pathologic fractures predominantly occur during non-traumatic everyday activities. The assessment of femoral mechanics in one-legged stance hase been previously used to study the incidence of spontaneous^27,28^ and pathologic fractures^21^, the behavior of the hip during daily activities^29^ or the load sharing between cortical and trabecular bone^30^. Initially, the stiffness was quantified by applying compressive load to induce axial displacement from 0 to 1 mm in all specimens in their intact condition. After creating a lesion in one specimen of each femoral pair, these specimens underwent additional QCT scanning for purposes of model generation, followed by another elastic test to assess the stiffness of the pathological bone. A second compression cycle was finally performed on all specimens until failure to determine the failure load.

### Homogenized voxel FE models

A homogenized continuum level voxel based FE model, previously developed for prediction of biomechanical behavior of the femur in different loading scenarios^16^, was adapted for the purpose of the present study. In short, QCT images were processed and voxels were directly converted into FE hexahedral model elements. The bone contour was segmented using the fill algorithm, as already described in previous studies for the human vertebrae^31^ and femora^16^. For each sample, the Hounsfield unit (HU) scale of the corresponding QCT image sequence was converted to the equivalent bone mineral density (BMD) scale (in mg HA/cm^3^) using a linear calibration law, computed from the two scales for the phantom inserts with known BMD values. The BMD range was limited to −100 and 1,400 mg HA/cm^3^ to restrict the effect of residual air and other artefacts. Due to the limited resolution of the QCT scans, no fabric measurement could be done and bone was therefore considered isotropic. The bone material was modelled using an isotropic elastic-damage constitutive law adapted^16^ from Garcia *et al*.^32^. Material nonlinearity was applied when bone was loaded beyond a yield limit, defined by a piecewise Hill criterion^33^, in which the inelastic behavior was driven by a damage scalar variable (between 0 and 1), that represents the reduction of the material elastic modulus^34^. The assigned material properties originate from preceding studies. Briefly summarized, multi-axial mechanical testing on a large number of human trabecular bone specimens was performed to assess the elastic and strength properties^35^. Due to the fact that those material parameters were measured in trabecular bone samples, a correction was defined for the poreless bone (BV/TV = *ρ* = 1) to provide the elastic modulus, compression ultimate stress, and tension ultimate stress for cortical bone^16^, considering previously published ultimate values^36,37^. Utilizing cement markers incorporated in the distal embedding, each specimen was positioned in a one-legged stance alignment, where the angle between the loading axis and the proximal shaft was equal to 20 degrees. This alignment was identical to the experimental condition. The images were cut proximally up to the polyurethane embedding. A polyurethane embedding layer (Poisson ratio: 0.3, Young’s modulus: 1.36 GPa) and a steel plate (Poisson ratio: 0.3, Young’s modulus: 210 GPa) were modelled isotropically at the cranial portion of the femoral head to exactly mimic the experimental setup^16^. The external bone contour was found with a filling algorithm, as already described in a previous study for the human vertebrae^31^ and femora^16^, followed by image coarsening to 3 mm × 3 mm × 3 mm voxel size. Image coarsening aimed to simulate QCT image resolution, requiring a minimum of radiation exposure in clinical routine. The FE mesh was generated by converting all voxels to hexahedral elements. In the next step the BMD scale was converted to a bone volume to total volume (BV/TV) scale with the linear equation BV/TV = 0.093*BMD + 1.077 as described previously^38^. Loading was imposed as vertical displacement of a reference node, located at the mean position of the most cranial voxel layer of the femoral head. All translations and rotations of the reference node except for vertical translation were unconstrained. Unconstrained in-plane translations should mimic the axle bearing used in the experimental setup, while unconstrained rotations should simulate motions between the proximal embedding and the femoral head. The translations of the nodes at the most distal portion of the shaft, starting at the polyurethane embedding, were fully constrained. Compression was simulated using a −5 mm vertical displacement of the reference node.

### Model simulation

**Figure 2:**
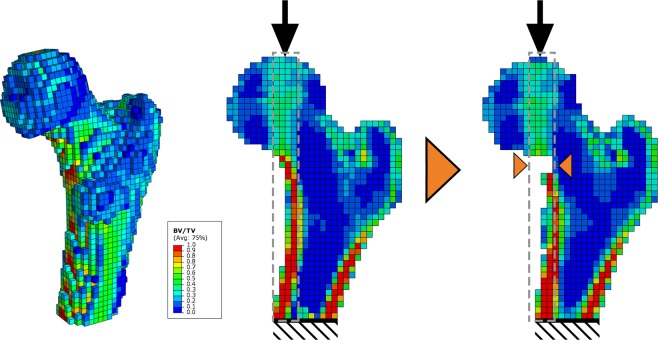
QCT-based finite element model with a voxel size of 3 mm × 3 mm × 3 mm in 3D and midplanes for intact- and specimen with a simulated inferomedial lesion (from left to the right). Specimen is positioned in one-legged stance. Values of highest BV/TV are in line with the vertical force vector (grey rectangle). Destroying the cortex in this region will result in redistribution of load and premature failure.

The generated QCT-based FE model (Fig. 2) had an average number of approximately 11,000 nodes and 13,000 elements. The models were generated in less then 30 minutes, including all necessary manual inputs. Following manual user inputs were needed: selection of four regions of interest: the proximal femur and three different phantom inserts, annotation of cement markers in the distal embedding, and selection of a threshold for the segmentation algorithm^39^. The models were solved on a conventional four CPU desktop PC with 3.4 GHz, in an average CPU time of 27 min with average necessary memory of 390 MB using Abaqus v.6.14-5. (Dassault Systemes Simulia Corp., Johnston, RI, USA).

Image processing, modelling and data evaluation were performed in a highly automated manner using the Medtool software.

### Statistical analysis

Pearsons product-moment correlation coefficient (R) was computed to investigate linear correlations between experimental and simulation data. Differences in mean stiffness and mean failure load between the three different groups (intact femora and femora with simulated lesions in two different sites) were investigated using the ANOVA and a post-hoc Tukey Honest Significant Difference Comparison. Differences with p-values lower than 0.05 were considered statistically significant. Each group was tested for normal distribution using Shapiro-Wilk test. The analysis was performed using IBM SPSS Statistics 24 (IBM Corp., Armonk, NY, USA).

## Results

### Stiffness and failure load

**Figure 3:**
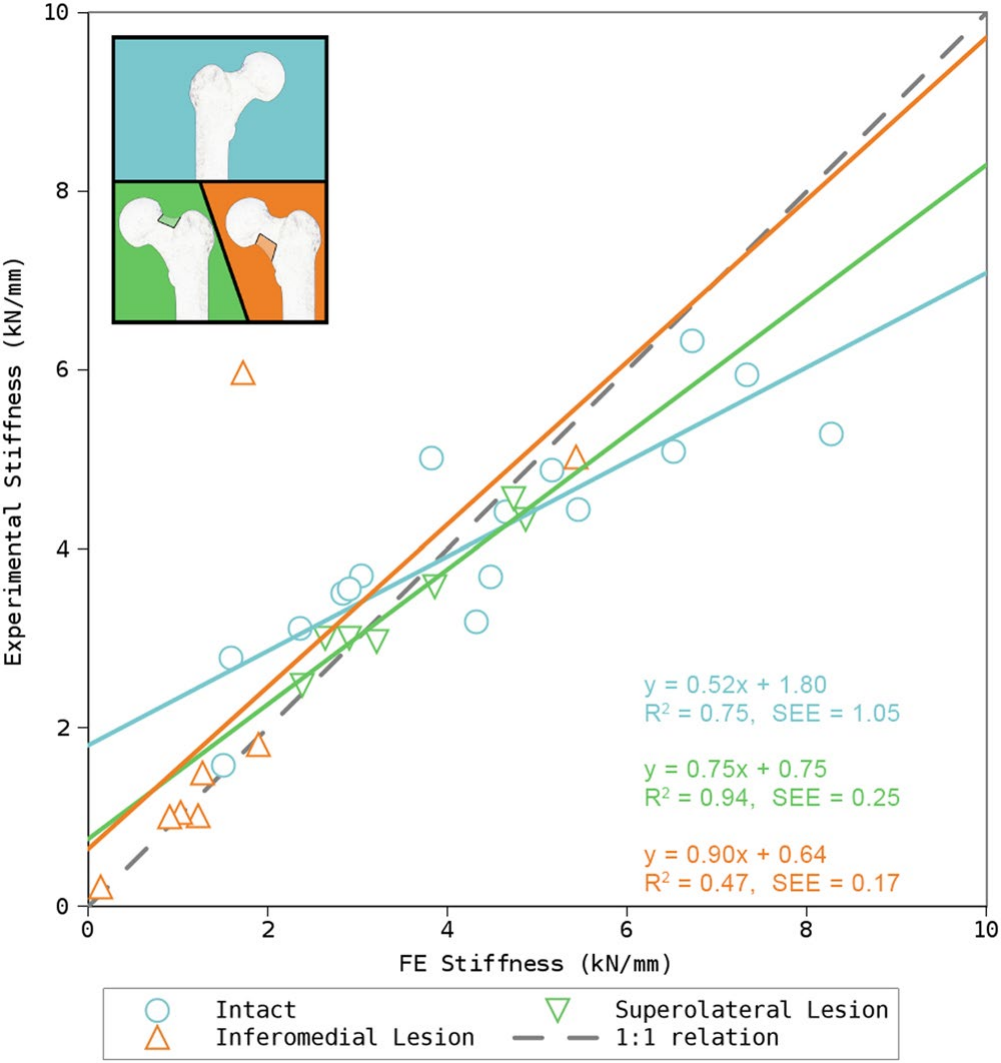
Results for prediction of stiffness in intact- and femora with simulated metastatic lesion in two different regions of the femoral neck. The ideal correlation (1:1 relation) is represented by the dashed line. The moderate correlation of femora with an inferomedial lesion is likely a result of a single outlier.

**Figure 4:**
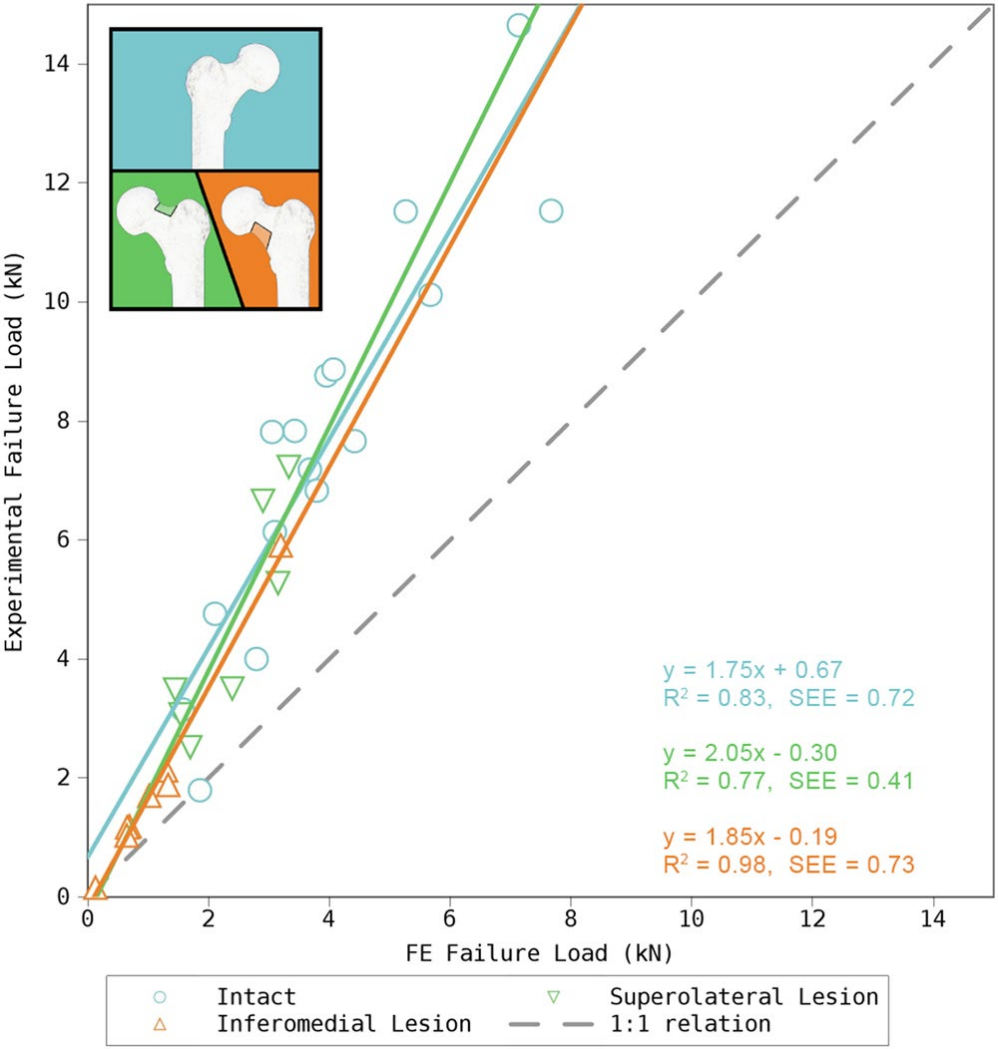
Results for prediction of failure load in intact- and femora with metastatic lesion in two different regions of the femoral neck. The ideal correlation (1:1 relation) is represented by the dashed line.

The correlations between the experimental and computed stiffness for the three groups: (1) intact-, (2) specimens with superolateral-, and (3) inferomedial lesion, were (1) R^2^ = 0.75 (p < 0.00005, 95% CI: 0.770–0.970), (2) R^2^ = 0.94 (p < 0.0005, 95% CI: 0.829–0.996), and (3) R^2^ = 0.47 (p = 0.058, 95% CI: −0.026–0.938), respectively. The correlations (1) and (2) were statistically significant. Strong correlations were found between the experimental- and computed failure loads in all three groups: (1) R^2^ = 0.83 (p < 0.00001, 95% CI: 0.77–0.97), (2) R^2^ = 0.77 (p < 0.01, 95% CI: 0.381–0.982), and (3) R^2^ = 0.98 (p < 0.00001, 95% CI: 0.966–0.998) (Figs 3 and 4). However, the failure load was underestimated approximately by a factor of 2. As expected a strong correlation was also found between the simulated stiffness and simulated failure load for all three groups ((1) R^2^ = 0.88 (p < 0.00001, 95% CI: 0.835–0.979), (2) R^2^ = 0.93 (p < 0.0005, 95% CI: 0.799–0.995), and (3) R^2^ = 0.98 (p < 0.00001, 95% CI: 0.955–0.998)).

The mean simulated stiffness was 4.44 ± 2.04 kN/mm (mean ± SD) (range: 1.50–8.27 kN/mm) for intact specimens, 3.52 ± 0.99 kN/mm (range: 2.47–4.55 kN/mm) for specimens with the superolateral lesion, and 1.70 ± 1.59 kN/mm (range: 0.14–5.43 kN/mm) for specimens with the inferomedial lesion. The mean simulated failure load was 3.91 ± 1.89 kN (range: 1.06–7.81 kN) for intact specimens, 2.35 ± 0.79 kN (range: 2.51–7.22 kN) for specimens with the superolateral lesion and 1.12 ± 0.92 kN (range: 0.12–3.20 kN) for specimens with the inferomedial lesion. A clear load maximum was seen in all load-displacement curves before the maximum displacement was reached. Fracture occurred at the simulated mean displacement of −1.40 mm in compression. Statistically significant differences were found between the mean stiffnesses of the intact- and specimens with the simulated inferomedial lesion (p < 0.005, 95% CI: 0.849–4.614) and between mean failure loads of intact- and specimens with the simulated superolateral and inferomedial lesion (p < 0.05, 95% CI: 0.045–3.201 and p < 0.0005, 95% CI: 1.347–4.362, respectively), which was in accordance with the experimental data.

### Fracture location

The QCT-based FE model predicted most fractures to occur in the subcapital region for intact specimens and in the femoral neck in specimens with simulated lesions. The model predicted the exact fracture location correctly in the majority of cases (21 out of 31 or in 68% of all specimens) (Table 1). The fracture location was correctly predicted in 56% of the intact specimens, in 100% in the superolateral-, and in 63% in the inferomedial-lesion group.

**Table 1:** Fracture locations recorded in the experiments and as predicted by the QCT-based FE model.

Specimen	Lesion	Fracture location	
		Experiment	FE
01R	superolateral	neck	neck
01L	intact	subcapital	subcapital
02R	superolateral	neck	neck
02L	intact	subcapital	neck
03R	superolateral	intertrochanteric	intertrochanteric
03L	intact	intertrochanteric	subcapital
04R	intact	subcapital	subcapital
04L	superolateral	subcapital	subcapital
05L	intact	subcapital	subcapital
05R	excluded specimen		
06R	intact	subcapital	subcapital
06L	superolateral	subcapital	subcapital
07R	intact	subcapital	subcapital
07L	superolateral	neck	neck
08R	intact	subcapital	subcapital
08L	inferomedial	subcapital	neck
09R	inferomedial	neck	subcapital
09L	intact	neck	subcapital
10R	inferomedial	neck	neck
10L	intact	subcapital	subcapital
11R	intact	subcapital	subcapital
11L	superolateral	neck	neck
12R	inferomedial	neck	neck
12L	intact	subcapital	subcapital
13R	inferomedial	neck	neck
13L	intact	subcapital	neck
14R	inferomedial	neck	neck
14L	intact	intertrochanteric	subcapital
15R	intact	subcapital	neck
15L	inferomedial	neck	neck
17R	intact	neck	subcapital
17L	inferomedial	subcapital	neck

### Correlation with vBMD, BMC, and CCD angle

Mean volumetric bone mineral density (vBMD) of all included specimens was 218.60 ± 53.70 mg/cm^3^ (range 82.97–336.56) and mean bone mineral content (BMC) was 35.39 ± 13.98 g (range 12.68–74.75). Neither the assessed densitometric (vBMD and BMC) nor geometric (CCD angle) parameters showed a significant correlation with the ultimate loads for any specimen group (Table 2).

**Table 2:** Pearson’s Correlation Coefficient R^2^ between volumetric bone mineral density (vBMD), bone mineral content (BMC), caput-collum-diaphyseal (CCD) angle, as well as failure load from simulation (FE Fu) and failure load from biomechanical experiments.

	vBMD (mg/cm^3^)	BMC (g)	CCD (degree)	FE Fu (kN)
Intact	0.75	0.68	0.15	0.83
Superolateral lesion	0.25	0.10	0.08	0.77
Inferomedial lesion	0.75	0.74	0.14	0.98

## Discussion

This study aimed to introduce a QCT-based finite element model to predict the biomechanical behavior of intact- and femora with metastatic lesions. To meet the demands of clinical applicability, the model was required to be easy to generate, fast to solve.

Simulation data provided statistically significant correlations for all biomechanical parameters, except for the stiffness in specimens with inferomedial lesions. This observation was very likely based on one single outlier (specimen 12R), which showed unusually high stiffness. While the stiffness was in range with other specimens in the simulation, it reached an unusually high value in the experiment. Possible causes could have been interlocking of the proximal setup, which, however, could have not been identified in recorded video or in video extensometer data or a not-visible pathologic alteration of bone tissue. Correlation of failure loads between models and experimental data was significant for all specimen groups. However, the failure load was underestimated by a factor of 2. These findings are similar to previously published data^16^, and can likely be attributed to limitations of coarsened QCT voxels in capturing the correct bone morphology. Luisier *et al*.^40^ showed a significantly improvement of fracture load prediction from QCT-based FE models using a high-resolution peripheral quantitative computed tomography (HR-pQCT)-based FE model and using a fabric-based orthotropic material model. However, given the strong and statistically significant validation of the QCT-based model, but also from clinical perspective these results alone are not sufficient to justify the higher radiation dose needed to reach such resolution in a central anatomical site, like the femur and in patients, who undergo frequent QCT-scanning due to rapid growth of the bone metastases. The authors of the present study also used rather large elements to reduce preprocessing and analysis time in order for the technique to be clinically applicable. Using such coarse models, the thin cortical shell cannot be resolved adequately, introducing geometrical artifacts and also discrepancies in fracture site prediction. It is important to stress that the bone material properties were obtained from previous research^35^ without any further internal calibration or tuning of the values. Even though tuning of the material properties would probably lead to improved predictions closer to the 1:1 relation, this was not the purpose of the present study. Nevertheless, the presented model showed strong correlation between the experimental and simulated data and proved its ability to reliably and fast predict the failure load for both the intact and the pathological femora. Despite the mentioned underestimation of the failure loads, the slopes of the regression lines were nearly identical for all three groups underlying the reliability of used methodology. In other words, structural variations (large geometrical changes) of bone due to metastases had limited influence on the model robustness. Pooled simulated data sets from all three groups correlated strongly with the experimental data (R^2 ^= 0.91 (p < 0.00001, 95% CI: 0.913–0.979)). Improved modelling approaches will be necessary to further increase the correlation to the experimental data. Alternatively, linear FE models (e.g.^17,20^) have shown promising potential for clinical applications because of their faster simulation and could possibly be useful in pathologic fracture load prediction as well. Nishimaya *et al*.^17^ reported mesh generation and simulation of a femur in less than twelve minutes. While studies using linear FE models have reported more favorable correlation to the experimental data, they also have not reached 1:1 relation, similar to the present study. Finally, despite their advantages and robustness, linear models still have to be applied and validated for pathological bone. Present data have confirmed the well known linear relationship between stiffness and ultimate load in intact bone. This observation was, however, not made for pathological bone and could affect the accuracy of linear FE models.

The experiments were able to show that simulated metastatic lesions in the femoral neck lead to reduction in both, stiffness and failure load and in addition that the site of the lesion had a great effect on the reduction magnitude. While the finite element analysis correspondingly found significant differences in failure load between groups with different sites of the metastases, all specimens would be considered for prophylactic surgery according to Mirels’ scoring system. Mirels’ scoring system combines four radiographic and clinical risk factors: degree of pain, lesional size or its cortical involvement, osteolytic versus osteoblastic nature, and anatomic location (upper limb, lower limb, peritrochanter). It represents the standard method for pathologic fracture prediction. Another common clinical methodology would not recommend any specimen for prophylactic stabilization, since less than 50% of its cortex were affected by the presented lesions^41–43^. The discrepancy between different clinical guidelines, as well as their insufficient sensitivity, again underlines the necessity for a more sensitive patient-specific solution.

As previously shown in biomechanical *in vitro* experiments^11^, a lesion in the inferomedial femoral neck produces significantly greater deterioration of biomechanical properties of the femur compared to a lesion in the superolateral neck. One-legged stance induces a considerable bending moment at the femoral neck^21^, with maximum value at the neck’s distal base. Also, the highest compressive stresses occur in the medial portion of the neck^44^. This results in the highest cortical thickness being present in the inferior neck, while the thinnest lies in the superior region (Fig. 2). Destroying the inferior cortex will eliminate the capacity of the femur to support the stress at the base of the medial femoral neck, while the remaining load must then be redistributed to adjacent regions, subjecting them to greater stresses, leading to premature failure. None of the established clinical methods for fracture risk prediction takes this effect into account^11^.

The present work has two major limitations. First, the use of QCT data, coarse voxel, and simplified model assumptions (isotropic bone material behavior, similar damage behavior for trabecular and cortical bone, isotropic damage, no softening in compression) have resulted in inaccurate failure load predictions in the hvFE model despite high correlations. However, there is still no reliable and clinically available imaging method to measure the trabecular orientation and bone anisotropy. The second limitation arises from the lesions, which were simulated by total bone removal and do not exactly represent metastatic geometries observed in clinical routine. Metastatic lesions often affect multiple bone regions and the cortex by spreading from the bone marrow. The aim of this study was not the perfect match with an individual pathology but the systematic validation of the QCT-based FEA for representative anatomies, pathologies, and loading conditions. Simulating metastatic lesions by total bone removal is common in the literature^11,23,24^, but it assumes the lesion to be fully osteolytic, affecting the total width of the cortical bone and would represent the clinical worst case scenario. Osteolytic lesions have poor mechanical properties, justifying their simulation by total bone removal, while at the same time there are no data on material properties of mixed or purely osteoblastic lesions. Further research is required on this topic. The focus of the present study was mainly the destruction of the cortex, which was shown to be subjected to highest strains^44^ in one-legged stance and which contributes to over 90% of bone’s strength^30^. In addition, high age of the donors does not adequately represent patients, who typically suffer a pathologic fracture.

In conclusion, this study showed the ability of the subject-specific QCT-based FE model to predict of the biomechanical behavior for both, the intact and pathological femur. Considering the usage of QCT data with low level of complexity and moderate computational resources needed for analysis performance and the high level of automation applied in this study, as well as the strong correlation with experimental data, the presented model could qualify as a good candidate for clinical applications. In order to make this model clinically applicable as a decision support system in prediction of pathologic fractures its reliability has to be evaluated in *in situ* scanning conditions for various loading scenarios. Finally, the image segmentation and model generation should be performed fully automated.
